# Heavy Metal Accumulation and Phytoremediation Potentiality of Some Selected Mangrove Species from the World’s Largest Mangrove Forest

**DOI:** 10.3390/biology11081144

**Published:** 2022-07-29

**Authors:** M. Belal Hossain, Zobaer Masum, M. Safiur Rahman, Jimmy Yu, Md. Abu Noman, Yeasmin N. Jolly, Bilkis A. Begum, Bilal Ahamad Paray, Takaomi Arai

**Affiliations:** 1 Department of Fisheries and Marine Science, Noakhali Science and Technology University, Noakhali 3814, Bangladesh; zobaermasum12@gmail.com; 2 School of Engineering and Built Environment, Griffith University, Brisbane, QLD 4111, Australia; jimmy.yu@griffith.edu.au; 3 Chemistry Division, Atomic Energy Centre Dhaka (AECD), Bangladesh Atomic Energy Commission, Dhaka 1000, Bangladesh; safiur.rahman@dal.ca (M.S.R.); jolly_tipu@yahoo.com (Y.N.J.); bilksab@baec.gov.bd (B.A.B.); 4 State Key Laboratory of Biogeology and Environmental Geology, China University of Geosciences, Wuhan 430074, China; abu.noman.nstu@gmail.com; 5 Department of Zoology, College of Science, King Saud University, P.O. Box 2455, Riyadh 11451, Saudi Arabia; bparay@ksu.edu.sa; 6 Environmental and Life Sciences Programme, Faculty of Science, University Brunei Darussalam, Jala Tungku Link, Gadong BE1410, Brunei; takaomi.arai@ubd.edu.bn

**Keywords:** heavy metals, sediments, mangroves, Sundarbans, phytoremediation

## Abstract

**Simple Summary:**

Plant or nature-based cleanup or removal of pollutants is one of the most promising eco-friendly approaches for sustainable ecosystem management. Consequently, the contamination level, accumulation and remediation ability of three mangrove plants (*Excoecaria agallocha*, *Avicennia officinalis*, *Sonneratia apetala*) and their surrounding sediments were studied. Analyses of accumulated metals by using several indices reveals that the area is low to moderately contaminated, and all the three plants examined can be used as phytoextractors as they have the ability to store metals in their tissues. *E. agallocha* can be used as a good phytostabiliser for Mn which can reduce the risk of erosion and leaching of this pollutant to water bodies. Furthermore, for metal extraction, *A. officinalis* was found more suitable than other two plants. Overall, the results indicate that these mangrove plants can be used in the phytoremediation of contaminated soils by metals.

**Abstract:**

Toxic metal pollution is a global issue, and the use of metal-accumulating plants to clean contaminated ecosystems is one of the most rapidly growing ecologically beneficial and cost-effective technologies. In this study, samples of sediment and three mangrove species (*Excoecaria agallocha*, *Avicennia officinalis*, *Sonneratia apetala*) were collected from the world’s largest mangrove forest (along the Northern Bay of Bengal Coast) with the aim of evaluating metal concentrations, contamination degrees, and phytoremediation potentiality of those plants. Overall, the heavy metals concentration in sediment ranged from Cu: 72.41–95.89 mg/kg; Zn: 51.28–71.20 mg/kg; Fe: 22,760–27,470 mg/kg; Mn: 80.37–116.37 mg/kg; Sr: 167.92–221.44 mg/kg. In mangrove plants, the mean concentrations were in the order of *E. agallocha* > *A. officinalis* > *S. apetala*. The mean (± SD) concentration of each metal in the plant tissue (root) was found following the descending order of Fe (737.37 ± 153.06) > Mn (151.13 ± 34.26) > Sr (20.98 ± 6.97) > Cu (16.12 ± 4.34) > Zn (11.3 ± 2.39) mg/kg, whereas, in the leaf part, the mean concentration (mg/kg) of each metal found in the order of Fe (598.75 ± 410.65) > Mn (297.27 ± 148.11) > Sr (21.40 ± 8.71) > Cu (14.25 ± 2.51) > Zn (12.56 ± 2.13). The contamination factor (CF) values for the studied metals were in the descending order of Cu > Sr > Zn > Fe > Mn. The values of I_geo_ (Geo-accumulation index) and CF showed that the area was unpolluted to moderately polluted by Zn, Fe, Mn, Cu and Sr. Enrichment factor (EF) values in both sampling stations portrayed moderate to minimum enrichment. Phytoremediation potentiality of the species was assessed by bio-concentration factor (BCF) and translocation factor (TF). BCF values showed less accumulation for most of the heavy metals (<1) except Mn which was highly accumulated in all mangrove plants. The translocation factor (TF) values depicted that most of the heavy metals were strongly accumulated in plant tissues (>1). However, the BCF value depicts that Mn was highly bioconcentrated in *E. agallocha*, but the translocation on leaves tissue were minimum, which reveals that *E. agallocha* is phytoextractor for Mn, and accumulated in root tissues. All the examined plants can be used as phytoextractors as they have bioconcentration factors <1 and translocation factors >1. However, *A. officinalis* is clearly more suitable for metal extraction than *S. apetala* and *E. agallocha* in terms of hyper-metabolizing capabilities.

## 1. Introduction

Ecosystems contaminated with heavy metals pose serious threat to environmental and human health [1]. Because these heavy metals are toxic, persistent, bio-accumulative, not naturally biodegradable and hence, can enter the food chain [2,3]. Chemical treatments are relatively successful for degrading organic contaminants in order to solve the issue of environmental pollution on the one hand, but they are quite expensive and environmentally unfriendly on the other. In addition, this technique is ineffective in removing toxic heavy metals from the soil [2]. Therefore, it is still in need of the development or use of effective, affordable and ecofriendly technology to solve the issue. The idea of using green plants to remove or reduce the metal contaminants, known as phytoremediation, has been successful as a promising environmental technology. This approach has been used for a long time in various developed nations, including the United States and Australia. However, due to a lack of comprehensive and reliable regional information, these strategies have yet to be widely used in developing nations.

Mangrove sediments are thought to sequester toxic metals as several studies have demonstrated that reforestation has enhanced reduction of metals from water and surrounding environments [4,5]. Large amounts of wastes containing metals are discharged into the coastal ecosystems such as mangroves through various channels as a result of growing industrialisation and urbanisation [4,5]. Heavy metals discharged into coastal ecosystems as a result of human activities are frequently associated with particulate matter, which settles and becomes deposited in sediments [6]. Therefore, coastal and estuarine systems are the important sinks for metals and land-derived pollutants [7]. However, heavy metal accumulation and distribution in sediments are substantially determined by mineralogical composition, sediment structure and physical transit [7]. Heavy metals at high concentrations in sediment are available to be absorbed by organisms and retained in their tissues, influencing biological responses and eventually hampering growth and development mechanisms [8]. As a result, coastal sediments are regarded to be important indicators for determining the health of ecosystems [9].

Mangroves are one of the most productive ecosystems on the planet, offering a wide range of ecosystem services such as animal feeding and habitat, erosion mitigation and coastal landform stabilisation [10]. These special plants or salt marshes grow in coastal sediments, serve as a medium of biological absorption and can change the rate of heavy metal adsorption for phytoremediation purposes [7,11]. These mangroves are also characterised as “green barriers” because of their exceptional capacity to reduce metal transmission to nearby environments [12]. Furthermore, the remarkable capacity of mangroves to survive in high-salt, anoxic environments, as well as their great resistance to heavy metal stress [13], contribute to their potential use in preventing anthropogenic poisons from diffusing into aquatic sediments [14]. The Sundarbans mangrove forest is one of the world’s largest and most complex and active ecosystems [15]. On a local and global scale, this area is significant from an ecological and economic standpoint. This mangrove habitat, however, is one of the most vulnerable tropical ecosystems, having received significant anthropogenic pollutant inputs due to its proximity to urban expansion [16] from shipping, tourisms, industrial wastewaters and agricultural pesticides, containing heavy metals [17].

Phytoremediation is a plant-based technique that uses plants to eliminate or reduce the bioavailability of elemental contaminants in the soil [18,19]. Phytoremediation, which takes advantage of plants’ unique ability to concentrate elements and chemicals from the environment and metabolise diverse molecules in their tissues, has a lot of potentiality for removing contaminants from the environment, particularly heavy metals [20]. Some techniques are now being utilised to remove these sorts of pollutants from the environments. But a large number of them are expensive and distant from their ideal performance. Such as, the chemical methods produce a huge amount of residues and increase the cost. Besides, both the chemical and thermal methods are difficult to handle, costly and also debase the important part of sediments. Phytoremediation, on the other hand, is a solar-driven, cost-effective, safe and environmentally sustainable remediation approach for removing toxins that may be used in situ [2,21].

Currently, there are several types of phytoremediation practices, such as, phytoextraction, phytodegradation, phytostabilisation and phytovolatisation. In the phytoextraction process, plants take up the substances from the sediment and store them on their cells. In the process of phytodegradation, plants convert the pollutants to nontoxic ones. In phytostabilisation, plants make the contaminants less bioavailable through releasing chemicals and binding it with the pollutants, whereas phytovolitisation makes the plants to take up the pollutants and release it as gas [11,22]. Since most plant roots are found in the sediment, they can play a key role in metal removal through phytoremediation, particularly phytoextraction and phytostabilisation, through filtration, adsorption and cation exchange, as well as plant-induced chemical changes in the rhizosphere [23,24]. As a result, it is critical to use native mangrove plants for phytoremediation because they are more naturally adapted to survive, grow and reproduce in stressful settings than plants transplanted from other ecosystems [7]. In spite of their importance, very few studies in Bangladesh examined the phytoremediation capability of mangroves to remove heavy metals [7,25]. Several studies have looked at the heavy metal contamination in the sediment of Sundarbans mangrove forests of Bangladesh part [26,27,28]. However, the phytoremediation potential of mangrove plants to remove metal contaminants from the sediment of Sundarbans mangrove forest is yet to be studied. Therefore, given the importance of mangroves and their phytoremediation abilities, the aims of this study are: i) To determine the concentration of heavy metals in mangrove sediment and plants; ii) to evaluate the degree of metal contamination in the study area; and iii) to estimate the accumulation and translocation ability of heavy metals in selected mangrove plants. The hypothesis is mangrove plants are highly potential to remove or accumulate heavy metals. The findings will help to identify the best candidate species for phytoextraction and/or phytostabilisation in the field.

## 2. Materials and Methods

### 2.1. Study Area

The coastal region of Bangladesh covers almost 29,000 km^2^ or about 20% of the country [29], whereas around 5% of them are naturally growing mangroves. Total mangrove area in Bangladesh is about 635,586 hectares, of which 618,586 hectares is naturally growing (Sundarbans, Chakaria Sundarbans and scattered mangroves) and 17,000 ha is manmade mangrove (coastal afforestation) [30]. The Sundarbans is the largest contiguous tidal halophytic mangrove ecosystem in the world, and also recognised as a UNESCO World Heritage Site [31]. The average annual rainfall in this area varies from 2000 to 1600 mm, and during the rainy season (June to September) maximum rainfall occurs. The higher temperature occurs during the March to June (26–34 °C), whereas in December to February the temperature falls to 12–25 °C. The relative humidity ranges from 70–80% annually [32]. However, the south-westerly monsoon wind blows over the area from the middle of March to end of September [32]. Along with these features, twice a day tidal inundation and huge sediment deposition due to the extensive network of rivers and channels with Sundarbans, promote this area as one of the most productive ecosystems of the country [32]. However, this mangrove forest is under risk because of anthropogenic activities like oil spillage, heavy metals and agrochemicals those might have affected this mangrove ecosystem [33].

**Figure 1:**
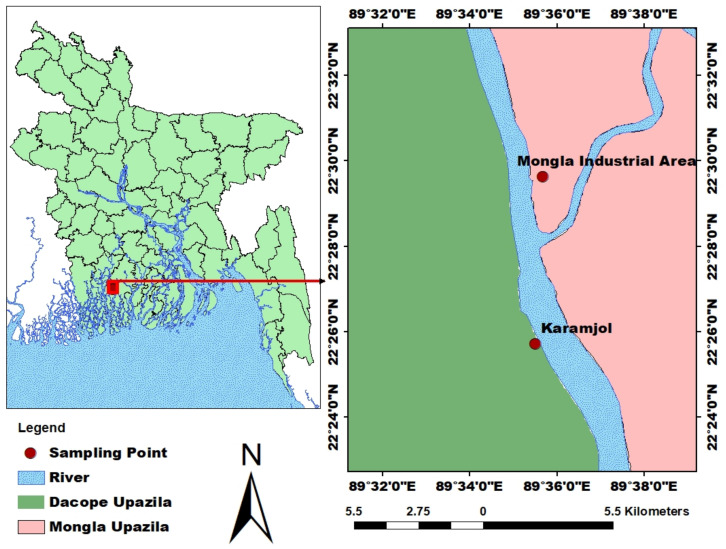
Map of the study area. Two sampling stations are shown in red dots.

The samples were collected from the Mongla industrial area (Station 1) located between 22.509862° N, 89.586971° E and Karamjol (Station 2) located between 22.428529° N, 89.590031° E under the upazila Mongla, Bangladesh (Figure 1). Mongla is a Sundarbans area in Bagerhat district and located at the bank of Pashur river. It lies between 22°33′ and 21°49′ North latitudes and between 89°32′ and 89°44′ East longitudes. Both sampling areas receive regular tidal inundation through the River Passur. The Sundarbans, the world’s biggest mangrove forest and one of the last remaining homes for rare Royal Bengal Tigers, are accessible by Karamjol (Station 2). Thousands of visitors from all over the world flock to Karamjol, a deep-in-the-forest ranger station that also acts as a crocodile and deer breeding centre.

### 2.2. Sample Collection, Preparation and Analysis

Sediment and plants samples were collected from 14 November to 16 November 2020 during low tide period with minimal disturbance. A total of six surface sediment samples (three replicate samples from each station) were taken from top 0–10 cm (recently deposited sediment), which covers an area of 1 m^2^. A composite sampling technique was followed. Moreover, three species of mangrove trees were selected for collecting samples, namely *Excoecaria agallocha*, *Avicennia officinalis*, *Sonneratia apetala*. These species were selected because of their dominancy in the study area and have not studied yet in Bangladesh for their phytoremediation potentiality. Without posing any detrimental effect to the plant, 36 plant samples (leaves and roots) were obtained from adult trees of similar age. Trees taller than 1 m and with an ambit of more than 20 cm at chest height were considered [11]. Leaves and roots were cut off the tree with a sharp sterilised knife, thoroughly rinsed to remove any clinging dirt and placed in a zip lock plastic bag before being transported to the laboratory.

After collection, the sediment samples were sieved in the laboratory with a plastic sieve to remove debris and vegetable matter. After that, each sample was placed in separate porcelain plate. Each dish containing the specific sample was placed in a 70 °C oven until a consistent weight was achieved. Using a mortar and pestle, the dry bulk of each sample was pulverised to a fine powder and stored in a plastic vial with the identification mark inside a desiccator. Finally, a pellet maker (Specac, Orpington, UK) employed the homogenous powder to form pellets under 10-ton pressure for elemental analysis by energy dispersive X-ray fluorescence (EDXRF, Epsilon 5, PANalytical, Almelo, The Netherlands) [34].

The dried plant samples were crushed to a fine powder using an agate mortar, and pellets were created using a CARVER type manual pelletizing machine at a pressure of 3 tons for powdered plant samples. The pelletised sample was placed on the X-ray fluorescence (XRF) system’s sample holder, and the sample was irradiated with the EDXRF Spectroscopy System. The target sample was excited with a Cd109 point source with a 22.4 KeV X-ray beam, resulting in the emission of characteristic X-rays, which were detected by the [Si (Li)] detector (Canberra) with a resolution of 175 eV at 5.9 keV, amplified by the spectroscopy amplifier and processed by the multi-channel analyser MCA (6K+channel). The constituents in the sample are determined qualitatively and quantitatively using the commercial software AXIL loaded on the computer [35].

### 2.3. Quality Control and Accuracy

Using the same approach as the experimental samples, the standard reference materials (marine sediment, IAEA 433, Austria) were examined to provide data quality assurance and quality control (QA/QC). For this investigation, the precision was usually 3–5%, depending on the RSD percent (relative standard deviation percent) of the samples and the relative error for standard reference materials. The accuracy of the standard reference materials was determined to be less than 5%, with a recovery rate of 94–106 percent. However, as extra material, the data for this study’s quality assurance and quality control (QA/QC) can be accessed (Table S1).

### 2.4. Ecological Risk Assessments of Heavy Metals

Several indices were used to assess the contamination state of sediment, including enrichment factor (EF), contamination factor (CF) and geo-accumulation index (I_geo_). The level of contamination in this investigation was compared with the average shale values suggested by Turekian and Wedepohl [36].

#### 2.4.1. Enrichment Factor (EF)

The following equation was used to calculate EF values which gives an idea about the influence of anthropogenic activities on metal concentration in the sediment [37]:

(1) $$\mathrm{Enrichment}\;\mathrm{Factor}\;(\mathrm{EF})\;=\;\frac{\left[M_{x}/{\mathrm{Fe}}_{x}\right]}{\left[M_{\mathrm{ref}}/{\mathrm{Fe}}_{\mathrm{ref}}\right]}$$

where, $[M_{x}$/${\mathrm{Fe}}_{x}]$ is the ratio of targeted metal concentration and Fe in sediment samples, and $[M_{\mathrm{ref}}$/${\mathrm{Fe}}_{\mathrm{ref}}]$ refers to ratio of the background value of the target metal and Fe. Iron (Fe) is reflected as the proxy or normalizing element [37]. Samples with an EF value greater than 1.5 are thought to have come from human activities [38]. On the basis of the degree of pollution, five contamination types are recognised [39]: minimal enrichment (EF < 2), moderate enrichment (2 ≤ EF < 5), very high enrichment (20 ≤ EF < 40) and extremely high enrichment (EF ≥ 40).

#### 2.4.2. Contamination Factor (CF)

To assess the extent of contamination of heavy metals, contamination factor and pollution load index have been applied [40].

The contamination factor (CF) parameter is expressed as:

CF = C_metal_/C_background_ (2)

where CF is the contamination factor, C_metals_ is the concentration of pollutant in sediment C_background_ is the background value for the metal. The metal enrichment in the sediment is reflected in the CF value. The geochemical baseline values in continental crust averages of the trace metals is under discussion. Taylor and McLennan [41] described the composition and evolution of continental crust, and the metal concentration stated by them used a background values for the metal in this study. The CF was classified into four groups [42], where the contamination factor CF < 1 refers to low contamination; 1 ≤ CF < 3 means moderate contamination; 3 ≤ CF ≤ 6 indicates considerable contamination and CF > 6 indicates very high contamination.

#### 2.4.3. Geo-Accumulation Index (I_geo_)

The I_geo_ value can evaluate the level of heavy metal contamination in sediment samples. The following equation, devised by Muller [43] is used to calculate it:

(3) $$I_{\mathrm{geo}}\;=\;\log_{2}\left[\frac{C_{n}}{1.5{\;B}_{n}}\right]$$

where C_n_ represents the heavy metal concentration in samples and B_n_ represents the heavy metal content in the geochemical background. The factor 1.5 is used to account for variances in background values caused by lithological differences. The level of contamination found in various sediments and soils is indicated by I_geo_. According to Muller [43], I_geo_ values are classified into seven classes: practically unpolluted (I_geo_ < 0), unpolluted to moderately polluted (0 < I_geo_ < 1), moderately polluted (1 ≤ I_geo_ < 2), moderately to strongly polluted (2 ≤ I_geo_ < 3), strongly polluted (3 ≤ I_geo_ < 4), strongly to extremely polluted (4 ≤ I_geo_ < 5), extremely polluted (I_geo_ ≥ 5).

### 2.5. Assessment of Phytoremediation Potentiality

The ability of local plants in the study area to withstand and accumulate heavy metals could be used for phytoextraction and bioremediation of the metal-contaminated station. In contrast, BCF and TF can be used to estimate a plant’s phytoremediation capacity [44]. Pollutants accumulate in the plant because the increased contaminants it absorbs are not processed fast [45]. The potential of native plants to undertake phytoremediation can be determined by comparing their bioconcentration factor (BCF) and translocation factor (TF).

BCF was calculated using the following two equations to determine the phytoextraction capabilities of the plants investigated [46].

(4) $${\mathrm{BCF}}_{\mathrm{leaf}}\;=\;\frac{C_{\mathrm{leaf}}}{C_{\mathrm{sediment}}}$$

(5) $${\mathrm{BCF}}_{\mathrm{root}}\;=\;\frac{C_{\mathrm{root}}}{C_{\mathrm{sediment}}}$$

TF was calculated using the following equation, which was adapted from the literature, to evaluate a plant species’ phytoremediation capabilities [44,47].

(6) $${\mathrm{TF}}_{\mathrm{leaf}}\;=\;\frac{C_{\mathrm{leaf}}}{C_{\mathrm{root}}}$$

where, the trace metal concentrations in the leaf and root, respectively, are represented by $C_{\mathrm{leaf}}$ and $C_{\mathrm{root}}$ and the metal concentration in sediment are represented as $C_{\mathrm{sediment}}$.

### 2.6. Statistical Analysis

The significant differences of heavy metal concentrations in soil and plant tissues were analysed by analysis of variance (ANOVA). Before, ANOVA test, homogeneity of variance and normality of data set were tested using Levene’s test and Shapiro–Wilk using Microsoft Excel 2007 (Microsoft, Redmond, DC, USA) and PAST (version 3; NHM, Norway). A probability of 5% was considered as significant. The linear regression with the metal concentration of sediment, plant roots and leaves were analysed to identify the relationship between them. The graphical representation of the study area was plotted using ArcGIS platform (version 10.3) and metal analyses using Graph Pad (Dotmatics, CA, USA; version 7).

## 3. Results and Discussion

### 3.1. Metal Concentrations in Sediment

**Figure 2:**
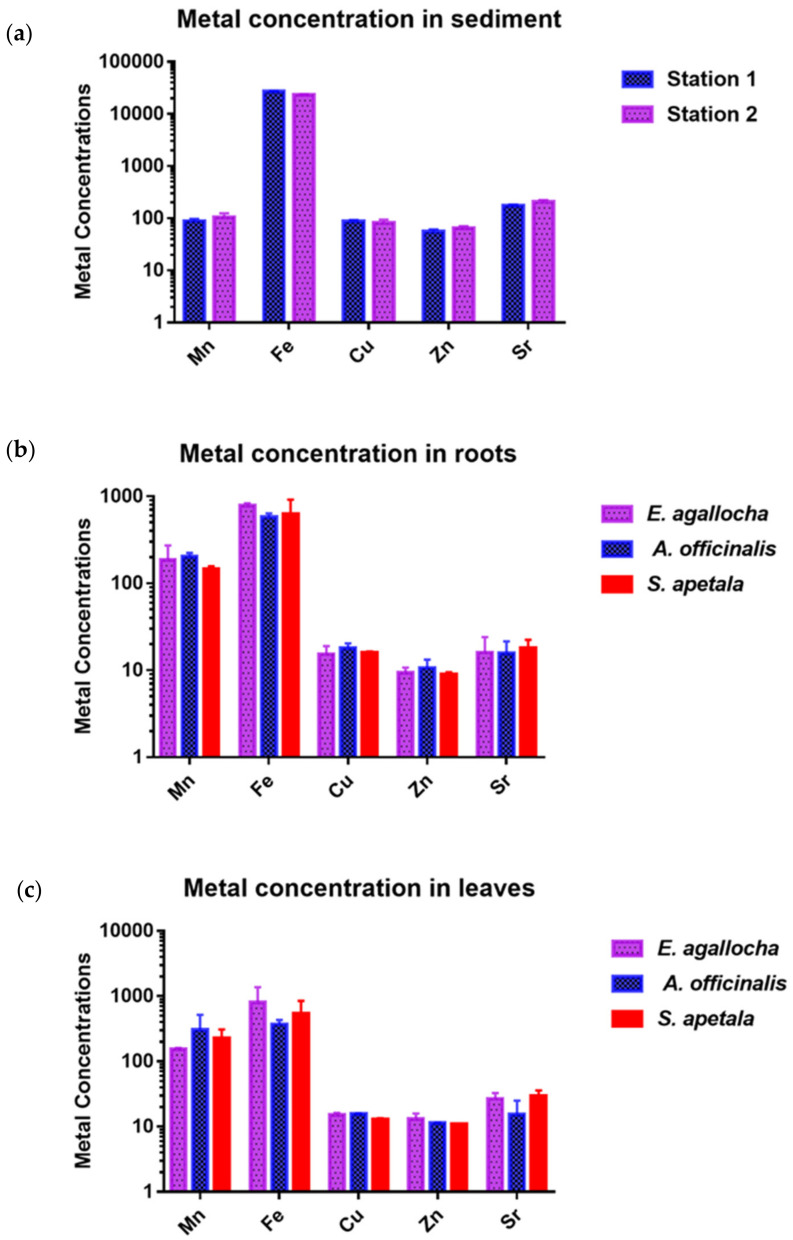
Metal concertation (mg/kg) in the sediments (**a**), roots (**b**) and leaves (**c**) of three mangrove species. Figure 2b and c present average values for both stations. Error bars indicate standard deviation.

The present study analysed five metals (Cu, Zn, Fe, Mn, Sr) from the mangrove sediments. Concentrations of heavy metals ranged as follows; Cu: 72.41–95.89 mg/kg; Zn: 51.28–71.20 mg/kg; Fe: 22,760–27,470 mg/kg; Mn: 80.37–116.37 mg/kg; Sr: 167.92–221.44 mg/kg (Figure 2a and Table 1). The average concentrations of the studied heavy metals in both stations showed the decreasing order of Fe (26,930 ± 478.2) > Sr (173.28 ± 21.45) > Mn (88.77 ± 16.27) > Cu (86.83 ± 9.39) > Zn (55.19 ± 7.32). Metal concentrations in the sediment of two stations was not significantly varied (F = 0.4962, *p* > 0.05). However, Cu, Fe and Zn concentrations were found higher in Mongla than the Karamjol area possibly the area was receiving the discharge of recently established industries such as oil refineries, coal and cement. Among the studied metals Fe, Zn, Cu and Mn were commonly studied in most of the similar previous studies, hence considered for comparing with their findings (Table 1). In our study, the Cu concentrations in both statins were much higher than the NOAA guidelines [48] and the ASV value of Turekian and Wedepohl [36]. Moreover, this finding of Cu concentration was higher than mangrove sediments in Kerala mangrove ecosystem [49], Pichavaram mangrove forest [50], Mahanadi delta mangrove area [51], Indian Sundarbans [25] and previous findings from Mongla area [26]. The higher level of Cu in the study area might be the result of anthropogenic activities such as vehicle and coal combustion emissions, car lubricants and natural phenomenon such as metal contents of rocks and parent materials, processes of soil formation [26]. The average concentrations of Mn in our study were 88.78 mg/kg in the Mongla area, and 103.97 mg/kg in Karamjal area. These findings are well below the ASV value and other relevant previous studies (Table 1). Even in Mongla Sundarbans area, Rahman et al. [26] found the average concentrations of Mn was 548 mg/kg, which is way higher than the findings of the present study (Table 1). Moreover, in the north-west coast mangrove sediment [52], and in Indian Sundarbans [25] the concentration of Mn was higher than our finding (Table 1). The concentrations of Fe seem similar to the previously recorded value from the Mongla area [26]. Though the concentration of Fe was far below than the ASV value, it was higher than the recorded value from Pichavaram mangrove forest [50], Indian Sundarbans [25] and North-West coast mangrove sediment of South America [52]. Similar to Fe, the concentrations of Zn are similar to the finding of previous study from Mongla Sundarbans area [26]. These concentrations of Fe obtained from the Mongla and Karamjal mangrove in the present study were lower than the ASV value [36], Kerala mangrove ecosystem [49] and Mahanadi delta mangrove area [51], but higher than Indian Sundarbans [25] and Pichavaram mangrove forest [50] (Table 1). The precipitation of Fe as iron sulphide, which is prevalent in mangrove habitats, could explain the high Fe concentrations. Iron is the primary metal that precipitates with sulphidic compounds in anaerobic sediments, and these sulphides serve as a key metal sink in the mangrove ecosystem [25].

### 3.2. Ecological Risk Assessment in Sediment

#### 3.2.1. Contamination Factor (CF)

In the present study, CF values of Mn, Fe, Zn, Sr were <1, which indicate low contamination rate in the sediment. In terms of Cu the values of CF are >1, but below 3, which denotes moderate contamination of sediment. The CF values for all heavy metals are in the decreasing order of Cu > Sr > Zn > Fe > Mn. The mean values of contamination factor of Cu, Mn, Fe, Zn, Sr are 1.86, 0.12, 0.53, 0.58, 0.63 (Table 2).

#### 3.2.2. Geo-Accumulation Index (I_geo_)

While assessing the toxicity of the metal contamination, the geoaccumulation index (I_geo_) is applied to assess the contamination of each metal on the sediment. The I_geo_ index was used to calculate the metal contamination levels in the Mongla and Karamjal station. It is divided into seven categories, ranging from unpolluted to very polluted. The I_geo_ grades for the sediments in the study area vary from metal to metal and from station to station (across metals and stations). In this study, sediment quality for Cu ranged from unpolluted to moderately polluted (0 ≤ I_geo_ < 1). For Fe, Mn, Sr concentrations, sediment quality was found practically unpolluted (I_geo_ < 0). I_geo_ values in all metals showed the decreasing order of Cu > Zn > Sr > Fe > Mn in both stations (Table 2).

#### 3.2.3. Enrichment Factor (EF)

The enrichment factor (EF) is a useful tool for determining the amount of pollutants in the environment [54]. Normalised EF values were determined using the continental shale abundance of Fe (6.75 %) as a benchmark [36] as well as using the average concentration of iron in the lower part of the studied cores. The mean EF for Cu, Mn, Fe, Zn, Sr were 3.52, 0.22, 1.0, 1.09, 1.18 respectively. In the current study, EF value for Cu found above 2 in both sampling stations suggests moderate enrichment in the area and the rest of the metals (Mn, Fe, Zn, Sr) have EF values < 2, which indicate deficiency to minimal enrichment in the area. Samples which have enrichment factor value > 1.5 is generally considered as indicative of human influence [38] (Table 2).

### 3.3. Concentration of Metals in Mangroves

Plants can absorb trace metals through their roots, branches and leaves and store them in various plant parts. Furthermore, the distribution and accumulation of trace metals are influenced by plant types, metal sources and sediment metal concentrations [55]. There is a wide range of trace metal uptake and distribution in the tissues of three mangrove species, which could be owing to the complex physiological mechanisms involving cell wall immobilisation, humic substance complexes and the presence of a barrier at the root epidermis [56]. In the examined three mangrove species, the mean (±SD) concentration in station 1 of each metal in the plant tissue (root) was found following the descending order of Fe (737.37 ± 153.06) > Mn (151.13 ± 34.26) > Sr (20.98 ± 6.97) > Cu (16.12 ± 4.34) > Zn (11.3 ± 2.39) mg/kg (Table S2). Whereas, in station 2, the respective metal levels were found in the order of Fe (571.57 ± 202.73) > Mn (207.13 ± 68.76)> Cu (15.18 ± 2.76) > Sr (12.28 ± 6.54) > Zn (9.50 ± 0.84) mg/kg (Figure 2b, Table S2). In the leaf part of the selected species, the mean concentration (mg/kg) of each metal in station 1 was found in the order of Fe (598.75 ± 410.65) > Mn (297.27 ± 148.11) >Sr (21.40 ± 8.71) > Cu (14.25 ± 2.51) > Zn (12.56 ± 2.13), whereas in station 2 the respective metal levels were found in the order of Fe (377.43 ± 74.37) > Mn (160.92 ± 25.29) > Sr (26.36 ± 12.22) > Cu (15.06 ± 2.76) > Zn (11.08 ± 0.69) (Figure 2c, Table S2). In terms of metal concentration in plants, the average concentration in the plants was in the order of *E. agallocha* > *A. officinalis* > *S. apetala* (Figure 2). However, the heavy metals in the roots of *A. officinalis* and *S. apetala* are almost similar, but in *E. agallocha* it was much higher comparatively. In leaves, the maximum metals were found in the *E. agallocha* plants, followed by *S. apetala* and *A. officinalis* (Figure 2b,c, Table S2). Particularly, Fe concentrations were higher in the roots of *A. officinalis* and *S. apetala* than *E. agallocha*; however, for Mn the pattern is reverse for the studied species. The concentrations of Cu and Zn were almost similar in roots and leaves, but for the Sr the concentrations were higher in leaves than the roots of all plants. ANOVA results showed the Fe and Mn concentrations in sediments and among mangrove species significantly varied (*p* < 0.05); however, for the rest of the metals the concentrations were not significantly varied specially among the plants (*p* > 0.05).

The concentrations of most accumulated heavy metals in mangrove tissues were higher in most cases than their respective concentrations in mangroves worldwide [25,57,58,59,60,61] (Table S3). The concentration of Fe in mangrove tissue of all the plants was the highest and Mn showed the second highest concentrations than the other heavy metals, which is very similar with the findings of Chowdhury et al. [25] in mangroves of India. *S. apetala* showed higher accumulation of Fe and Mn than in *E. agallocha* and *A. officinalis* which is consistent with Chowdhury et al. [25]. The concentration of metal (Zn, Mn, Sr) was higher in leaves than the roots for all the studied species except the concentrations of Cu and Fe which were higher in roots of these mangroves. The variation of specific metal accumulation depends on their individual physiological rhythms and prevailing ecological conditions of the inhabiting environment [59,60]. For example, in acid-sulphate soil, the Fe concentrations are higher and resulted in higher accumulation of this metal in mangrove plants [25].

### 3.4. Phytoremediation Potentiality of Mangroves

#### 3.4.1. Bioconcentration Factor (BCF)

**Figure 3:**
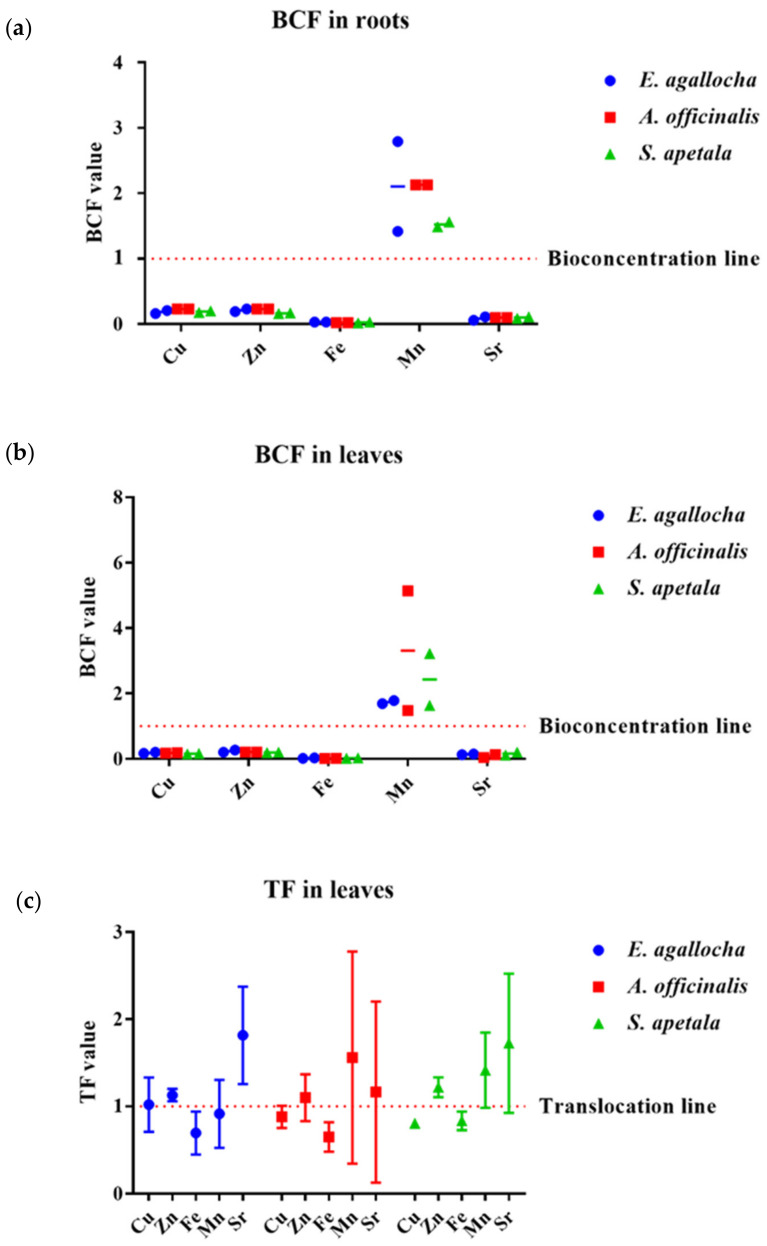
Bioconcentration factor (BCF) in roots (**a**) and leaves (**b**) and translocation factor (TF) estimated (**c**) for the heavy metals in the three mangrove species. BCF and TF were based on the average metal concentration for both stations. The values below the bioconcentration and translocation line indicate the species are less suitable for phytoextraction and translocation of metals.

The bioconcentration factor (BCF) from sediment to various body parts (root and leaf) of three mangrove species (*Excoecaria agallocha*, *Avicennia officinalis* and *Sonneratia apetala*) was used as an indicator of species accumulation ability from nature, and is calculated as the proportion of metal concentration in plant tissue and sediment. The values of BCF for Fe, Cu, Zn, Mn and Sr metals from sediment to roots and leaves of the mangrove species were calculated in this study (Figure 3a,b). The results showed that all the plant organs (roots and leaves) had BCF values < 1 for the elements Fe, Cu, Zn, Sr. However, the BCF value > 1 for Mn was found in both roots and leaves of all three mangrove species. Overall, the highest BCF value (5.14) was found for Mn in the leaf of *Avicennia officinalis*, whereas the lowest BCF value was found in *Sonneratia apetala*. The BCF value for Cu in all three species was found in the following descending order *A. officinalis* > *E. agallocha* > *S. apetala*. For Zn, highest BCF value was found in *E. agallocha* and lowest BCF value was found in *A. officinalis*. The BCF value for Zn was found in the following descending order *E. agallocha* > *S. apetala* > *A. officinalis*. For Fe, the highest BCF value was found in *E. agallocha* and lowest BCF value was found in *S. apetala*. The BCF value for Fe was found following descending order *E. agallocha* > *A. officinalis* > *S. apetala*. For Mn, highest BCF value was found in *Avicennia officinalis*, lowest BCF value was found in *Excoecaria agallocha*. The BCF value for Mn was found in the following ascending order *A. officinalis* > *S. apetala* > *E. agallocha*. The highest and lowest BCF values for Sr were found in *Excoecaria agallocha* (Figure 3, Tables S2 and S4). The BCF value for all metals except Mn for all the plants was found <1 which indicated the unsuitability of these plants as phytoextraction process. BCF of Mn was >1 possibly due to the stimulation of growth factors for the mangrove species. Agoramoorthy et al. [58] and Chowdhury et al. [25] also reported same BCF value for mangrove plants in their studies in India.

#### 3.4.2. Translocation Factor (TF)

The translocation factor (TF) is required for a detailed explanation because the BCF does not sufficiently describe the entire scenario of metal accumulation in the plant body. Figure 3c and Tables S2 and S3, show the TF of Cu, Zn, Fe, Mn and Sr for *E. agallocha*, *A. officinalis* and *S. apetala* based on metal concentration ratios in the leaf and root of mangrove species. In this study, the translocation factor (TF) varied between the plant parts, and the non-essential metals such as Sr had the higher TF values than the TF values of essential metals in the studied mangroves. The average translocation of metals from root to leaf was found to be in the order of Sr > Mn > Zn > Cu > Fe. The highest and lowest translocation factors of mangroves were found 1.30 in *Sonneratia apetala* and 0.91 in *Avicennia officinalis* for Zn, 0.91 in *Sonneratia apetala* and 0.52 in *Excoecaria agallocha* for Fe, 2.42 in *Avicennia officinalis* and 0.64 in *Excoecaria agallocha* for Mn, 2.29 in *Sonneratia apetala* and 0.43 in *Avicennia officinalis* for Sr and 1.24 for *Excoecaria agallocha and* 0.79 in *A. officinalis*. TF values were found >1 for maximum metals except Fe and Cu in the studied plants which mean these plants can actively take up trace metals from the sediment and are able to accumulate them in their aerial parts, as a result can be good phystabilisers. This result was very similar with the investigation of Chowdhury et al. [25] of Indian mangroves.

#### 3.4.3. Relationship between Metal Concentrations in Sediment and Plants Tissue

**Figure 4:**
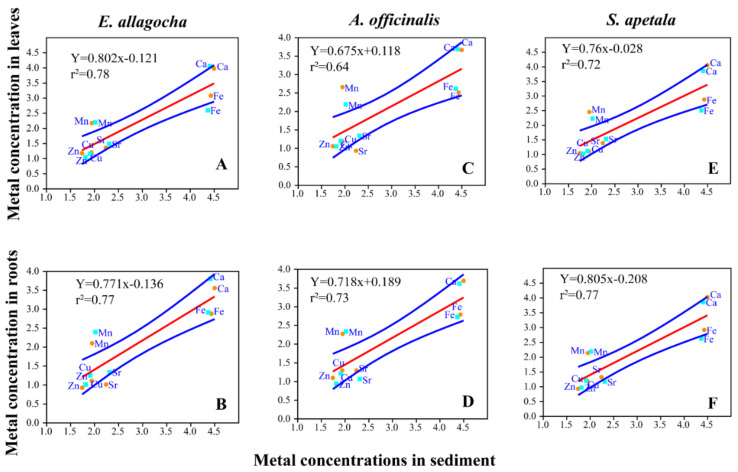
Linear regression model between metal concentration in sediment with the metal concentration in leaves (**A**,**C**,**E**) and roots (**B**,**D**,**E**) of three mangrove plants (*E. allagocha*—**A**,**B**; *A. officinalis*—**C**,**D**; *S. patella*—**E**,**F**).

The linear regression model shows the relationship between the metal concentration in sediment and plant tissue (Figure 4). Strong significant positive correlations depict the strong relationship between the metal concentrations in sediment and plant tissues. The linear regression between the log transformed metal concentrations in sediment and mangroves roots showed a strong positive correlation. Similarly, the regression between sediment’s and leave’s metal concentration also showed similar strong positive relationship like roots. The high values of correlation coefficients of determination (r^2^ = 0.64 to 0.78) for sediments and plant tissue clearly indicate the association of metals in sediments and plant tissues. The lowest correlation value (r^2^ = 0.64) was found for the leaves of *A. officinalis* and the highest for *E. agallocha.* The roots of all plants showed higher values of co-efficient of determination indicating significant higher association (*p* = 0.0003). However, the regression of *S. apetala* and *E agallocha* had negative intercept, whereas *A. officinalis* had positive intercept in both roots and plants regression analysis with sediment.

In our study, though we found that Mn is bioconcentrated in every species, highest bioconcentration was found in *A. officinalis.* Similarly, the translocation of metals in the leaves *A. officinalis* was higher than other two mangrove species. In addition, the regression model also indicates the phytoremediation capability of *A. officinalis* is higher than *S. apetala* and *E. agallocha*. However, all their species showed almost similar types of responses and relationship towards the metals in sediment. On the contrary, the *E. agallocha* species had the bioconcentration value higher than 2, but the translocation value less than 1, which depicts that *E. agallocha* possesses the ability to store them in their root tissues that was not present in the other two species. Therefore, we speculate that all three mangrove species examined in this study have good capability of phytoremediation, but the *A. officinalis* species would be the best fit for phytoextraction and *E. agallocha* species might be used for phytostabilizing heavy metals from the sediment of the Sundarbans area of Bangladesh.

#### 3.4.4. Practical Implications of This Study

As the most unavoidable and alternative ways of terrestrial soil reclamation, phytoextraction and phytostabilisation have received a lot of attention. Mangrove plants are very well studied as the major player in removing metal contaminants from the sediments and most of the species possesses the ability of phytoextraction or phytostabilisation. Therefore, the comparative study of various native mangroves helps to identify the useful candidate for metal phytoextraction or phytostabilisation from the mangrove sediments. These findings will help environmental managers and policy-makers to use local mangrove plants to remediate contaminated lands and to reduce the threat to mangrove ecosystem. However, to get a comprehensive knowledge, long-term monitoring studies and phytoremediation potentiality of other mangroves are recommended.

## 4. Conclusions

This study described the levels of five heavy metals in the sediments of Sundarbans mangrove area, contamination degrees in the environment and the potentiality of three native plants to limit the pollutant through phytoremediation. The concentrations of most metals in this mangrove sediment were found to be higher (except Mn) than in other mangrove sediments from around the world. The concentrations of Cu and Fe were found to be higher than the background value indicating anthropogenic contamination by these specific metals. The contamination factor (CF), geoaccumulation index (I_geo_) and enrichment factor (EF) revealed that the sediments were largely unpolluted to moderately polluted by copper (Cu), and practically unpolluted by the other metals (Fe, Mn, Zn, Sr). With the exception of Mn, the plant species *E. agallocha*, *A. officinalis* and *S. apetala* had BCF values less than one, indicating a weak ability to accumulate heavy metals. However, in the case of TF, all of the plants exhibited values greater than one for the majority of the metals, indicating that these plants can translocate metals from root to leaf and may operate as a phytoremediator in the study region. The BCF, TF and linear regression models show that *E. agallocha* species has a stronger potential for phytoextraction, whereas *A. officinalis* could be used as a heavy metal phytoextractor in this area.

## Figures and Tables

**Table 1:** The average concentrations of common trace elements (mg/kg) in the sediment of present study and selective mangrove wetlands around the world.

Location	Cu	Mn	Fe	Zn	Sr	References
Mongla, Sundarbans	86.82 ± 5.57	88.78 ± 8.40	26,930 ± 478.2	55.18 ± 5.6	173.08 ± 8.7	Present study
Karamjal, Sundarbans	81.13 ± 12.85	103.97 ± 20.5	23,357 ± 516.9	55.11 ± 5.36	204.05 ± 19.1	Present study
Mongla, Sundarbans	18.22	548	26,720	53.13	-	[26]
North-West coast mangrove sediment, South America	139.46	359.06	13,431.1	331.31	-	[52]
Kerala mangrove ecosystem, southern part India	76.73	-	-	127.6	-	[49]
Pichavaram mangrove forest, south eastern India	46	25	1770	25	-	[50]
Mahanadi delta mangrove area, India	17.9	-	37,810	98.3	-	[51]
Indian Sundarbans, West Bengal	36.03	709.06	11,097	40.42	-	[25]
Mangrove ecosystems from Senegal, West Africa	3.5	21	-	5.4	-	[53]
Average Shale Value (ASV)	45	850	47,200	95	-	[36]
Threshold Effects Level (TEL)	18.7			124		[48]

**Table 2:** Assessment of pollution of heavy metals in the sediment of Mongla (Station 1) and Karamjal (Station 2) mangrove sediments.

	Concentration Factor (CF)		Enrichment Factor (EF)		Geo-Accumulation index (I_geo_)	
Metals	Station 1	Station 2	Station 1	Station 2	Station 1	Station 2
Cu	1.93	1.8	3.38	3.64	0.363	0.27
Mn	0.11	0.13	0.18	0.25	−3.85	−3.62
Fe	0.57	0.49	1	1	−1.39	−1.6
Zn	0.58	0.58	1.018	1.17	−1.37	−1.37
Sr	0.68	0.58	1.19	1.17	−1.41	−1.38

## Data Availability

Data are available on request.

## Supplementary Materials

The following supporting information can be downloaded at: https://www.mdpi.com/article/10.3390/biology11081144/s1, Table S1: Analytical results obtained on certified reference materials (mg/kg) and the limit of detection (LOD) of the instrument (EDXRF, Epsilon 5, PANalytical, The Netherlands), Table S2: Metal concentrations in the root and leaves of three mangrove plants, Table S3: A comparison of data on heavy metal concentrations (mg/kg) in mangrove plant reported from other mangrove ecosystems of India, Saudi Arabia and present study, Table S4: Bioconcentration factor (BCF) and Translocation factor (TF) values in the root and leaves of three mangrove plants.
