# Supplementary material for: Heavy Metal Accumulation and Phytoremediation Potentiality of Some Selected Mangrove Species from the World’s Largest Mangrove Forest
Source: Biology (Basel). 2022 Jul 29;11(8):1144. doi: 10.3390/biology11081144 (PMC9405028; doi:10.3390/biology11081144)
Supplement: Supplementary file 1 [file biology-11-01144-s001.zip › biology-1689703-supplementary.pdf]

sustainability

MDPI

Article

Heavy metal accumulation and phytoremediation potentiality of some selected mangrove species from the world's largest mangrove forest

M. Belal Hossain[1,2,*], Zobaer Masum[1], M. Safiur Rahman[3], Jimmy Yu[2], Md. Abu Noman[4], Yeasmin N. Jolly[3], Bilkis A. Begum[3], Bilal Ahamad Paray[5] and Takaomi Arai[6]

1 Department of Fisheries and Marine Science, Noakhali Science and Technology University, Noakhali, 3814, Bangladesh; belal.hossain@nstu.edu.bd; zobaermasum12@gmail.com
2 School of Engineering and Built Environment, Griffith University, Brisbane, QLD 4111, Australia; jimmy.yu@griffith.edu.au
3 Chemistry Division, Atomic Energy Centre Dhaka (AECD), Bangladesh Atomic Energy Commission Dhaka 1000, Bangladesh, safiur.rahman@dal.ca, jolly_tipu@yahoo.com, bilksab@baec.gov.bd
4 State Key Laboratory of Biogeology and Environmental Geology, China University of Geosciences (Wuhan), Wuhan 430074, PR China, abu.noman.nstu@gmail.com
5 Department of Zoology, College of Science, King Saud University, PO Box 2455, Riyadh, 11451, Saudi Arabia bparay@ksu.edu.sa
6 Environmental and Life Sciences Programme, Faculty of Science, Universiti Brunei Darussalam, Jalan Tungku Link, Gadong, BE 1410, Brunei Darussalam.; e-mail: takaomi.arai@ubd.edu.bn
* Correspondence (mbhnstu@gmail.com)

Table S1. Analytical results obtained on certified reference materials (mg/kg) and the limit of detection (LOD) of the instrument (EDXRF, Epsilon 5, PANalytical, The Netherland).

Element	LOD	Certified reference values	Values found	RE (%)	Recovery (%)
Cr	2.0	77.2	76.9	0.31	99.7
Mn	2.5	1180	1125	4.66	95.3
Fe	1.1	27100	26112	3.65	96.4
Cu	1.0	32.7	30.6	6.33	93.7
Zn	0.7	74.8	72.7	2.86	97.1
Ni	1.2	9.7	10.1	4.12	104.1
As	0.4	18.3	17.7	3.22	96.8
Pb	0.9	22.8	22.5	1.54	98.5

Table S2. Metal concentrations in the root and leaves of three mangrove plants

	E. agallocha		*A. officinalis*		*S. apetalla*	
	Station 1	Station 2	Station 1	Station 2	Station 1	Station 2
**Roots**						
Cu	125.9135	248.222	188.7745	218.5015	138.7165	154.6535
Zn	758.171	820.7805	619.392	532.5765	834.551	421.976
Fe	12.8345	17.9205	19.787	16.3735	15.763	16.32
Mn	8.467	10.339	12.5335	8.814	8.5165	9.3535
Sr	10.323	21.871	19.877	11.6745	21.1805	14.855
**Leaves**						
Cu	149.888	158.5095	456.2655	154.058	285.6475	170.195
Zn	1210.819	396.511	325.187	413.128	760.2575	322.6345
Fe	15.928	14.347	15.738	15.947	12.64	13.2925
Mn	15.0995	11.145	11.467	11.393	11.0745	10.674
Sr	22.7895	30.9915	8.6105	22.2315	24.5835	34.0455

Table S3: A comparison of data on heavy metal concentrations (mg/kg) in mangrove plant reported from other mangrove ecosystems of India, Saudi Arabia and present study

Site	Mangrove species	Plant tissue	Cu	Zn	Fe	Mn	Sr	Refereces
Red Sea Coast, Saudi Arabia	*Avicennia marina*	Root	40.02	18.27	3913.14	67.27	-	[57]
		Leaf	4.17	4.23	39.54	11.14	-	
Tamil Nadu, India	*Avicennia officinalis*	Leaf	14.8	107.8	225.3	87.8	-	[58]
Kerala, India	*Avicennia officinalis*	Leaf	101.7	119.5	678	579	-	[59]
Mongla, Bangladesh	*Avicennia officinalis*	Root	19.79	12.54	619.39	188.78	19.88	Current study
		Leaf	15.74	11.45	325.19	456.27	8.61	

Dhamra Estuary, India	*Phoenix Paludosa*	Root	36.05	33.41	129.78	168.08	-	[60]
		Leaf	8.21	18.49	257.82	29.89	-	
Sundarban Mangrove,India	*Sonneratia apetala*	Root	9.61	9.56	750.24	62.37	-	[25]
		Leaf	9.34	16.82	484.52	323.61	-	
Mongla, Bangladesh	*Sonneratia apetala*	Root	15.77	8.52	834.55	138.72	21.18	Current study
		Leaf	12.64	11.11	760.26	285.65	24.58	
West Bengal, India	*Excoecaria agallocha*	Root	NA	NA	NA	NA	NA	[61]
		Leaf	19.53	_	585.11	533.81	NA	
Mongla, Bangladesh	*Excoecaria agallocha*	Root	17.93	12.84	820.78	125.91	21.87	Current study

Kader, A.; Narayan Sinha, S. Heavy metal contamination in the sediment and plants of the Sundarbans, India. Chemistry and Ecology, 2018, 34(6), 506-518.

Table S4. Bioconcentration factor (BCF) and Translocation factor (TF) values in the root and leaves of three mangrove plants.

	E. agallocha		*A. officinalis*		*S. apetalla*	
	Station 1	Station 2	Station 1	Station 2	Station 1	Station 2
**BCF in roots**						
Cu	0.21	0.16	0.23	0.23	0.18	0.2
Zn	0.23	0.19	0.23	0.23	0.16	0.17
Fe	0.031	0.032	0.023	0.023	0.031	0.018
Mn	1.42	2.79	2.13	2.13	1.56	1.49
Sr	0.11	0.06	0.1	0.1	0.11	0.09
**BCF in leaves**						
Cu	0.17	0.2	0.18	0.19	0.15	0.16
Zn	0.27	0.2	0.21	0.21	0.2	0.19
Fe	0.027	0.017	0.012	0.018	0.028	0.014
Mn	1.69	1.78	5.14	1.48	3.22	1.64
Sr	0.15	0.13	0.04	0.13	0.12	0.2

TF in leaves						
Cu	0.8	1.24	0.79	0.97	0.8	0.81
Zn	1.18	1.08	0.91	1.29	1.3	1.14
Fe	0.87	0.52	0.53	0.77	0.91	0.76
Mn	1.19	0.64	2.42	0.7	1.72	1.11
Sr	1.42	2.21	0.43	1.9	1.16	2.29
